# Aflatoxin in Dairy Cows: Toxicity, Occurrence in Feedstuffs and Milk and Dietary Mitigation Strategies

**DOI:** 10.3390/toxins13040283

**Published:** 2021-04-17

**Authors:** Yun Jiang, Ibukun M. Ogunade, Diwakar Vyas, Adegbola T. Adesogan

**Affiliations:** 1 Department of Animal Sciences, University of Florida, Gainesville, FL 32611, USA; jiangyun0110@ufl.edu (Y.J.); diwakarvyas@ufl.edu (D.V.); 2 Division of Animal and Nutritional Science, West Virginia University, Morgantown, WV 26506, USA; ibukun.ogunade@mail.wvu.edu

**Keywords:** aflatoxin, dairy cows, mitigation strategies, sequestering agents

## Abstract

Aflatoxins are poisonous carcinogens produced by fungi, mainly *Aspergillus flavus* and *Aspergillus parasiticus*. Aflatoxins can contaminate a variety of livestock feeds and cause enormous economic losses, estimated at between US$52.1 and US$1.68 billion annually for the U.S. corn industry alone. In addition, aflatoxin can be transferred from the diet to the milk of cows as aflatoxin M1 (AFM1), posing a significant human health hazard. In dairy cows, sheep and goats, chronic exposure to dietary aflatoxin can reduce milk production, impair reproduction and liver function, compromise immune function, and increase susceptibility to diseases; hence, strategies to lower aflatoxin contamination of feeds and to prevent or reduce the transfer of the toxin to milk are required for safeguarding animal and human health and improving the safety of dairy products and profitability of the dairy industry. This article provides an overview of the toxicity of aflatoxin to ruminant livestock, its occurrence in livestock feeds, and the effectiveness of different strategies for preventing and mitigating aflatoxin contamination of feeds.

## 1. Introduction

Aflatoxin contamination is common in various food and feed ingredients [1,2,3]. The consumption of aflatoxin-contaminated diets by dairy cows, sheep or goats results in transfer of the toxin to milk, resulting in a human health hazard [4,5]. The potential economic losses to the U.S. corn industry by aflatoxin contamination were estimated at between US$52.1 and US$1.68 billion annually [6].

In addition to the huge economic impact to producers, aflatoxins are carcinogens, and, therefore, they pose a significant human health hazard. The U.S. Food and Drug Administration (FDA) has set an action level for aflatoxin M1 (AFM1) of 0.50 µg/kg in liquid milk, total aflatoxins of 20 µg/kg in feed ingredients offered to dairy cattle, 100 µg/kg for breeding cattle, 300 µg/kg for finishing beef cattle and 20 µg/kg in foods intended for human consumption [7]. The European Commission set up an action level for AFM1 of 0.05 µg/kg in liquid milk, AFB1 of 20 µg/kg in all feedstuffs, 10 µg/kg in complete feeds, and 5 µg/kg in complete feeds for dairy animals [8,9]. Outbreaks of aflatoxin poisoning have occurred in many countries across the world ranging from the first detection of the disease in England in 1960 when it was called Turkey “X” disease, to more recent outbreaks in dogs in the United states [10] and in humans in Kenya, India, Thailand and Tanzania [11,12]. From March to June 2011, more than 75 dogs died after consuming pet food contaminated with aflatoxins in the U.S. [10]. The most severe outbreak in Kenya resulted in 125 deaths in 2004 [13]. In addition, aflatoxin contamination of food was associated with underweight and growth impaired children [14,15].

In dairy cows, chronic exposure to aflatoxins can reduce performance, impair liver function, compromise immune function, and increase susceptibility to diseases [16,17]. In addition, aflatoxin B1 (AFB1) can also impair reproductive function by reducing viability and DNA integrity of bull sperm [18] and causing damage to the bovine preimplantation embryo [19]. Hence, strategies to lower aflatoxin contamination and the transfer of aflatoxin to milk are required for safeguarding animal and human health. This article provides an overview of the toxicity of aflatoxin to ruminant livestock, its occurrence in livestock feeds, and the effectiveness of different strategies for preventing and mitigating aflatoxin contamination of feeds. The review also recommends priority areas for future research.

## 2. Aflatoxin Types

**Figure 1:**
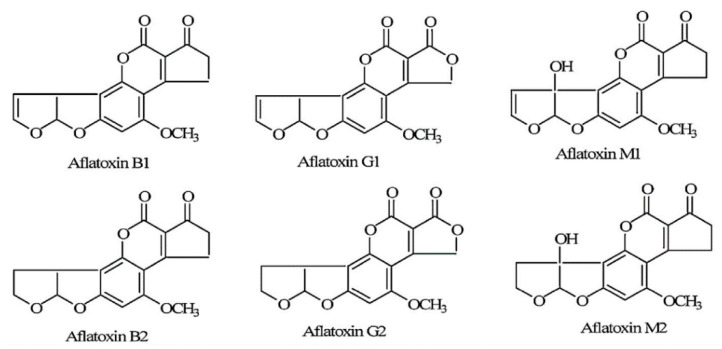
Chemical structure of aflatoxins B1, B2, G1, G2, M1 and M2 (Zhang et al., 2011) [21].

The major types of aflatoxin are B1, B2, G1, G2, M1, and M2 and their chemical structures are shown in Figure 1 [20,21]. Aflatoxin B and G groups have differences in their ring structures, properties in thin-layer chromatography, and have blue-green and green fluorescence, respectively [22]. Among these compounds, AFB1 and AFB2 are the most commonly occurring, which can be hydroxylated to AFM1 and AFM2, respectively, which are sometimes found in milk and dairy products [23]. Aflatoxin B1 can be transferred to its milk metabolite, AFM1 from various feedstuffs such as hay, silage, and grains, after mammals such as humans, dairy cows, sheep or goats, ingest contaminated feeds [5,24,25]. According to the International Agency for Research on Cancer (IARC), AFB1 is a Group 1 carcinogen to humans and AFM1 is classified in Group 2B as a possible carcinogen to humans [24]. Different animal species have various levels of susceptibility to aflatoxins. The median lethal dose (LD_50_) of AFM1 for rats is 1.5 mg/kg body weight (BW) while the LD_50_ of AFB1 for rats ranges from 1 to 18 mg/kg BW [26], whereas, for ducklings, turkey, poultry, rabbits, and pigs, it ranges from 0.3 to 0.6 mg/kg BW. Values of 2–5 mg/kg BW apply for sheep and 0.5–1.0 mg/kg BW for calves [27,28].

## 3. Occurrence and Prevalence

Several authors have reported the occurrence of aflatoxin in different feedstuffs and foods [29,30,31]. A study conducted during 2009–2010 by Rodrigues and Naehrer [29] reported that out of 4363 samples collected from the Americas, Europe, Oceania and Asia, 31% of samples were contaminated with aflatoxins (B1, B2, G1, G2) at an average concentration of 20 µg/kg for positive corn samples. The authors reported that mycotoxin contamination depends greatly on the region or sub-region where the contaminated crop is located, with those in the tropics and subtropics having greater concentrations than those in temperate areas. For example, 60% of samples were contaminated with aflatoxins with an average concentration of 43 µg/kg in South East Asia, while only 10% of samples were contaminated with an average concentration of 1 µg/kg in Australia and New Zealand. In some African countries, the prevalence and contamination level of aflatoxin seemed to be quite high according to several surveys, such as those in Kenya [30,31,32], although low aflatoxin contamination levels in feeds were also reported by surveys such as those conducted in Egypt and Tanzania [33,34]. A survey in Kenya reported mean AFB1 concentrations of 109, 89.7 and 196 µg/kg from feed samples collected from feed manufacturers, feed retailers, and farms [30]. Another survey in Rwanda tested 3328 feeds and feed ingredients samples collected from dairy farms, poultry farms, feed vendors, and feed processors and reported mean aflatoxin levels (B1 + B2 + G1 + G2) of 109, 44, 104 and 89 µg/kg, respectively [31]. In their samples, whole corn, corn bran, and mixed feed had the highest contamination levels at 161, 111, and 106 µg/kg, respectively.

Biomin Inc. (Ferndale, Michigan) conducted a worldwide survey of mycotoxin contamination in feed ingredients in 2018 and 2019 (Herzogenburg, Austria) and their results are summarized in Table 1 (Biomin Mycotoxin Survey, 2018 [35]) and Table 2 (Biomin Mycotoxin Survey, 2019 [36]). In 2018, approximately 37% of corn was contaminated with aflatoxins in Asia, 19% in South and Central America, 18% in Europe, and 9% in North America. The average aflatoxin concentration in positive samples was highest in Asia (42 µg/kg), followed by North America (36 µg/kg), and South and Central America (11 µg/kg). Asia and North America had aflatoxin concentrations above the FDA action level for positive feed samples (20 µg/kg). In the Biomin 2019 global survey, Biomin Inc. reported that over 25% of finished feed samples were positive for aflatoxin in Asia, Africa, South and Central America. Samples from the Middle East and North Africa had the highest mean concentration of 42 µg/kg while corn samples from Asia, the Middle East and North Africa, and South and Central America had the aflatoxin contamination rates of 31, 37 and 21%, respectively. Notably, corn samples from Asia had a 31% contamination rate with a mean concentration among positive samples of 43 µg/kg.

Most of the prior surveys have examined levels of aflatoxin B_1_ in feeds, but the risk of transfer of dietary AFB1 to AFM1 in milk and the associated health risks highlight the importance of studying the prevalence of aflatoxin contamination of milk. One of the most comprehensive compilations of published data on AFM1 levels in milk across the world was recently completed by Turna and Wu [37]. Surveys from most countries showed that at least a proportion of the milk had no detectable AFM1 levels, or AFM1 levels were detected below the EU action level of 0.05 µg/kg. However, some surveys from certain countries in Asia, the Middle East, South America, and Africa reported positive contamination in up to 100% of samples, and many of these had concentrations exceeding the FDA action level of 0.05 µg/kg AFM1. These authors reported that several nations including Pakistan, India, and several sub-Saharan African nations, had AFM1 levels in milk that substantially exceeded the United States and European Union regulatory limits for AFM1, indicating potential risk to individuals in those nations with high milk consumption.

## 4. Risk Factors for Contamination

In general, stressors such as temperature, drought, moisture, insect infestation, diseases, hail and other factors that can physically damage plants or interfere with the growth of plants can predispose crops to aflatoxin contamination [38,39,40]. In terms of weather, dry and hot conditions predispose growing plants with aflatoxin contamination, while warm and wet conditions favor the contamination after maturation [41]. The prevailing weather is an important determinant of aflatoxin contamination, with dry and hot weather, particularly prolonged droughts, predisposing crops to aflatoxin contamination [42]. This was evident from the record-breaking heat and drought in 2012, which resulted in atypically high aflatoxin (B1 + B2 + G1 + G2) contamination of feeds in the southern Corn Belt States in the U.S. [6]. In Texas, 60% of corn was contaminated with aflatoxin levels greater than 20 µg/kg during 2012, while less than 15% was contaminated at the same level in 2013 [6]. The Ohio Valley was estimated to have lost US$170–454 million due to aflatoxin contamination in 2012. A similar case was reported in Serbia after the prolonged drought of 2012, where 68.5% of samples were contaminated with aflatoxin with a mean concentration of 36.3 µg/kg, whereas no contamination was reported from 2009–2011 [42].

### 4.1. Temperature Stress

Studies have shown that aflatoxin production is highly temperature sensitive because temperature can affect the expression of aflatoxin biosynthetic genes [43,44]. Schindler et al. [43] tested the growth rate and aflatoxin-producing ability of two isolates of *A. flavus* at temperatures of 2 °C to 52 °C. Aflatoxin production was not related to the growth rate of *A. flavus* and one isolate had maximal growth of *A. flavus* at 41 °C with no aflatoxin production. Maximal aflatoxin production occurred at 24 °C and maximal growth of *A. flavus* at 29–35 °C. The type of aflatoxin produced and the ratio of AFB1 to AFG1 also varied with temperature. In temperatures below 7 °C or higher than 41 °C, there was no aflatoxin production even after 12 weeks of fungal growth. The different aflatoxin-producing rates at different temperatures may be due to modulation of the gene expression. OBrian et al. [44] showed that an isolate of *A. flavus* had maximum aflatoxin production at 28–30 °C but production decreased at temperatures close to 37 °C, which is the optimum temperature for growth of *A. flavus*. They observed that 103 genes, including all aflatoxin biosynthetic genes, were more highly expressed at 28 °C than 37 °C. Similarly, Liu et al. [45] reported all aflatoxin biosynthetic pathways genes of *A. flavus* were downregulated at 42 °C versus at 37 °C.

### 4.2. Drought Stress

Jones et al. [38] reported a significant correlation between AFB1 production and reduced crop yield and suggested that stress conditions that reduce yield may predispose corn to aflatoxin contamination. In their study, drought stress reduced yield and caused elevated AFB1 contamination, while irrigation reduced fungal infection and AFB1 contamination. They also reported that the cultivar and planting date combination that led to silking during periods of high airborne spore loads increased the aflatoxin contamination rate of kernels. Payne et al. [46] conducted a 4-year field study on reducing aflatoxin contamination in corn by irrigation and tillage. Corn under natural drought stress in North Carolina received normal or delayed irrigation. They showed that irrigation and subsoiling tillage reduced infection and aflatoxin production by *A. flavus*.

### 4.3. Diseases, Insects and Other Physical Damage

Payne et al. [46] reported that wound-inoculated corn had a more drastic increase in aflatoxin contamination than naturally infected corn during a drought. Even irrigation and subsoiling were not effective at reducing aflatoxin contamination in wound-damaged corn under drought stress. Queiroz et al. [5] reported that corn silage made from rust infected plants had aflatoxin contamination of 5200 µg/kg. A study by Hell et al. [47] showed that no aflatoxin was detected in insect damage-free corn sampled from four agroecological zones in 1993, while in the same year, a 30% contamination rate (mean 78 µg/kg) was observed in corn with >70% cobs damaged.

## 5. Effects on Ruminant Animals

### 5.1. Effects on Performance and Health

Dietary aflatoxin contamination can negatively impact animal performance. Queiroz et al. [5] fed 75 µg of AFB1/kg of diet dry matter (DM) to dairy cows and observed lower milk fat yield, 3.5% milk protein concentration and tendency of lower milk yield. Ogunade et al. [48] reported that feeding 75 µg of AFB1/kg diet DM to lactating dairy cows tended to reduce milk yield by 2.5 kg and reduced 3.5% fat-corrected milk yield by 1.7 kg. Harvey et al. [49] observed that consumption of 2.6 mg aflatoxin (type not specified) per kg of diet DM reduced BW gain of growing wether lambs. Similarly, Edrington et al. [50] reported that feeding growing lambs a diet containing 2.5 mg/kg aflatoxin (type not specified) reduced average daily gain, intake, and feed efficiency. However, some studies reported that aflatoxin did not impact dairy cow productivity. For instance, Sulzberger et al. [51] reported that 100 µg/kg of AFB1 did not affect milk production, intake, or feed efficiency. Similarly, Rodrigues et al. [52] showed that feeding 105.5 µg/kg aflatoxins (a mixture of B1, B2, G1 and G2) did not impact milk performance, intake, or efficiency.

The form of aflatoxin may influence the animal performance and health response. Many studies on aflatoxin effects use pure aflatoxin because it allows greater control of the dose applied and more contamination prevention. However, natural forms of the toxin may cause more severe damage due to the synergistic effects of different types of aflatoxin as well as other metabolites and their fungal source. For instance, Applebaum et al. [53] reported that 471 µg/kg of pure aflatoxin (AFB1) did not affect the milk production of dairy cows but 583 µg/kg of impure aflatoxin (AFB1 plus other aflatoxins and metabolites produced by culturing *Aspergillus parasiticus*) reduced milk production of dairy cows. Thus, studies conducted with pure aflatoxin B1 may underestimate the toxicity of aflatoxin to animals. Interestingly, in a study by Gallo et al. [54], a relatively low inclusion level, 17.53 µg/kg AFB1, relative to that in other studies with the pure toxin, reduced the intake and feed efficiency of dairy cows. This was possibly because of co-occurrence and synergies with other toxins since the contaminated corn meal feed was sourced from in-field crop inoculation with a mycotoxigenic *A. flavus* strain.

High aflatoxin concentrations (such as those above 2 mg/kg diet DM) can have severe impacts on animal growth, production and health. Several studies have observed altered concentrations of plasma metabolites in ruminant animals consuming diets contaminated with aflatoxin, which can indicate changes in immune response or compromised animal health [5,49,51]. For example, in the study by Sulzberger et al. [51], 100 µg/kg of AFB1 decreased plasma concentrations of aspartate aminotransferase and glutamate dehydrogenase in dairy cows, likely indicating alteration or suppression of liver function. Queiroz et al. [5] fed 75 µg of AFB1/kg of diet DM to dairy cows and observed an increased concentration of plasma haptoglobin and β-integrin, indicating an increased inflammatory response caused by aflatoxin. Recently, Ogunade et al. [48] reported that feeding 75 µg of AFB1/kg diet DM to lactating dairy cows reduced red blood cell count and hemoglobin concentration in the plasma. Harvey et al. [49] observed that consumption of 2.6 mg aflatoxin (type not specified) per kg of diet DM increased aspartate transaminase, gamma–glutamyl transferase activities, prothrombin time, and concentrations of cholesterol, uric acid, and triglyceride values and decreased concentrations of glucose, albumin, urea nitrogen and urea-to-creatinine ratio in the serum of growing wethers. Similarly, Edrington et al. [50] reported that feeding growing lambs a diet containing 2.5 mg/kg aflatoxin (type not specified) increased activities of aspartate aminotransferase and gamma–glutamyl transferase, total protein and cholesterol concentrations and decreased concentrations of several serum parameters such as alkaline phosphatase, albumin, inorganic phosphorus, iron, and total iron-binding-capacity. In their study, feeding aflatoxin also increased clotting time, hematocrit concentration, and white blood cell count. Fernandez et al. [55] reported that lambs fed 2 mg/kg aflatoxin (mixture of AFB1, B2, G1 and G2) for 37 d had reduced bacteriostatic activity of the serum and in vivo cellular immunity, suggesting that animals exposed to aflatoxin can be more susceptible to infectious disease.

### 5.2. Effects on Ruminal Fermentation and Microorganisms

The ability of aflatoxin to inhibit synthesis of DNA and RNA [56] and interact with enzymes may induce lower ruminal microbial activity when it is ingested by ruminants. The activated AFB1 metabolite and AFB1-8,9 epoxide can form a covalent bond with N7 guanine, and it forms adducts in cells, which leads to G-T transversion, DNA lesions, mutations, and tumors [57].

Several studies have investigated the effects of aflatoxin on the growth of selected ruminal microorganisms and ruminal fermentation [58,59,60]. Aflatoxin may decrease animal growth and performance by disrupting rumen microbial growth and function [58]. Sinha and Arora [59] reported that treating cotton cellulose incubated in rumen fluid in vitro with 100, 250, 500, 1000 and 1250 μg/kg of aflatoxin resulted in cellulose disappearance rates of 43.4, 20.9, 21.3, 15.5, and 16.4%, respectively. Furthermore, increasing the dose of aflatoxin reduced microbial protein synthesis from 48 to 31, 11, 10, 9 and −2.5 mg/20 mL, respectively. Westlake et al. [61] reported that 1000 and 10,000 μg/L of AFB1 inhibited in vitro DM degradation of alfalfa hay by 50 and 67%, respectively, and the effects were attributed to the toxic effects on ruminal microorganisms. Jiang et al. [60] reported that 320, 640, and 960 ng/mL of AFB1 did not affect in vitro DM disappearance in buffered-ruminal fluid but reduced gas production rate during fermentation of alfalfa or ryegrass hay. The highest dose (960 ng/mL) also reduced the total volatile fatty acids concentration by 12.7 and 9.6% when alfalfa or ryegrass hay were fermented in vitro, respectively [60].

However, it should be noted that such negative effects of aflatoxin on ruminal fermentation are attributable to high doses of aflatoxin (100 to 10,000 μg/L) in in vitro studies, which are less likely to occur in nature. In vivo conditions have more complex rumen environments, which may degrade and cause rapid absorption of aflatoxin into the blood stream [54], resulting in fewer effects on ruminal fermentation. Sulzberger et al. [51] reported that in lactating dairy cows, the peak ruminal AFB1 concentration was only 0.20 µg/L after challenging cows with 100 µg/kg/d of AFB1 for 3 d; however, the rumen fluid collection time relative to dosing aflatoxin was not specified. Jiang et al. [62] showed that 0.75 µg/L AFB1 did not affect in vitro rumen fermentation and digestibility of a dairy cow total mixed ration. Edrington et al. [50] reported that 2.5 mg aflatoxin per kg of diet DM reduced the intake, daily gain, and feed efficiency in growing lambs, and caused liver damage but had no effects on rumen fermentation. Similarly, Jiang et al. added 63 µg/kg AFB1 to the diet of lactating cows but found no negative effects of AFB1 on ruminal fermentation or the ruminal bacterial community [63,64]. These studies suggest that the negative impact of aflatoxin on animal performance is due more to the systematic toxicity effects, including immunosuppression, rather than direct toxicity to rumen microorganisms.

### 5.3. Effects on Reproduction

There is relatively little published information on aflatoxin effects on reproduction in dairy cows or bulls. Previous studies in other species have shown that exposure to aflatoxin can have a negative impact on various aspects of reproduction in both male and female animals. Some recent studies also confirmed the negative impact of aflatoxin on bull spermatozoa, fertilization competence and preimplantation embryo development in dairy cows [18,19,65]. Komsky-Elbaz et al. [18] reported that AFB1 reduced sperm viability of bulls, indicated by lower integrity of the plasma membrane, and it also reduced mitochondrial membrane potential and DNA integrity of sperm. A recent study by the same group showed that exposure of spermatozoa to 10 µM AFB1 resulted in differential expressions of 345 genes that are involved in cellular pathways, such as embryo and placenta development, cell cycle, DNA repair and histone modification and signaling pathways [65].

In addition, aflatoxin impaired oocyte and the preimplantation development of embryos by inducing overproduction of reactive oxygen species (ROS) [19]. The ROS, including mainly superoxide (O_2_∙−), hydroxyl radical (∙OH), and hydrogen peroxide (H_2_O_2_), are free radicals and are small molecules that disrupt cellular organelle function [66]. They can target macromolecules such as lipids, proteins, DNA and RNA and cause peroxidative damage to cells or even apoptosis [66]. A recent study by Jiang et al. [19] reported that AFB1 can inhibit development of preimplantation bovine embryos by reducing the percentage of oocytes becoming blastocysts partially through overproduction of ROS. However, antioxidant addition reversed overproduction of ROS but did not prevent the reduction in development to the blastocyst stage. The authors suggested that exposure to dietary aflatoxin may cause potential embryonic loss in dairy cows. The underlying mechanism by which AFB1 induces ROS production may be through suppression of superoxide dismutase and glutathione peroxidase activity, thus reducing antioxidant capacity [67]. In addition, ROS can be generated by many pathways including the metabolic processes of many xenobiotics by the cytochrome P450 system, which is known to metabolize AFB1 to a highly reactive metabolite, AFB1-8,9-epoxide [68].

Overall, aflatoxin can impair fertility of both female and male animals by affecting reproductive organs and cells and hinder embryonic development both pre- and post-implantation. In addition, aflatoxin exposure can impair immunity, reduce performance of animals, and is a food safety hazard when present in animal products. Thus, it is crucial to prevent or mitigate aflatoxin contamination of diets whenever possible.

## 6. Strategies for Preventing and Mitigating Aflatoxin Contamination

### 6.1. Preharvest Prevention

To prevent or reduce aflatoxin contamination, risk factors predisposing plants to aflatoxin contamination should be minimized, such as dry and hot conditions during growth, warm and wet conditions after maturation, drought, insects and diseases that can physically damage plants or interfere with their growth [38,40,41]. Good agronomic practices are essential for preventing aflatoxin contamination. For example, crops should be planted on time, harvested at the proper maturity and at the proper height to minimize fungal contamination from soil, adequately irrigated, appropriately treated with pesticides and herbicides to avoid physical damage to crops, stored under dry and cool conditions, and ensiled with antifungal inoculants to prevent aflatoxin contamination of silage [5,38,41].

Due to environmental concerns about overusing pesticides and herbicides, safer and more environmentally friendly biocontrol agents of aflatoxin have been developed in the United States, which includes non-aflatoxigenic strains of *A. flavus* to competitively exclude aflatoxin-producing *Aspergillus* species in the field [69,70,71]. A commercial biological control agent, AflaGuard, containing a harmless strain of *Aspergillus flavus* has been developed in the United States, while Aflasafe, containing a blend of four atoxigenic strains of *A. flavus*, was registered in Nigeria [72]. In a 10-year, large-scale study, Aflasafe had high efficacy when used for one year or multiple years in Nigeria [73]. They reported that grains from treated plots had over 80% less aflatoxin contamination than those from untreated plots. Over 95% of corn crops in plots treated with Aflasafe had contamination levels below the regulatory limit (20 µg/kg) and a significant portion contained less than the minimum level (4 µg/kg) of aflatoxins. A recent study conducted in Ghana showed that application of two aflatoxin biocontrol agents, Aflasafe GH01 and GH02, consistently reduced aflatoxin concentration by 99% on average in 800 maize and groundnut farmers’ fields during 2015 and 2016 [74]. The use of biocontrol agents for aflatoxin control is a good prevention strategy in the field especially in areas where aflatoxin contamination is a major concern or when good agronomical practices are difficult to achieve. Several excellent literature reviews about preventing aflatoxin contamination preharvest have been published [75,76], hence, the subject will not be discovered further here.

### 6.2. Post-Harvest Mitigation Treatments

#### 6.2.1. Ammoniation

Ammonia treatment can destroy aflatoxin by altering the molecular structure irreversibly after sufficient exposure. It can be applied in gaseous form, in solution or with substances that release the gas [77]. Gardner et al. [78] reported that gaseous ammonia treatment reduced aflatoxin contamination of peanut meal from 121 µg/kg to levels that were not detected, cottonseed meal from 350 to 4 µg/kg, and cottonseed meal from 519 µg/kg to below 5 µg/kg. Samarajeewa et al. [79] reviewed 27 studies that used ammoniation to inactivate aflatoxins in feedstuffs and reported a 90% reduction in aflatoxin levels in most studies regardless of the ammonia concentration (0.5–6.7%), form (gas, anhydrous, NH_4_OH), pressure (<1 bar to 3.1 bar), temperature (ambient, and up to 145 ℃), duration (15 min, hours, or days), and substrates (corn, peanut meal, cotton seed meal, cottonseed). Ammoniation has also reduced the transfer of AFB1 from contaminated diets to milk AFM1. Fremy et al. [80] reported that feeding lactating dairy cows rations containing ammonia-treated aflatoxin-contaminated peanut cake instead of untreated contaminated (1100 µg/kg of AFB1) cake reduced milk AFM1 concentration from 28 µg/kg (2.6% AFM1 of the ingested AFB1) to below 0.1 µg/kg. Nevertheless, ammoniation is not widely used to detoxify dairy cow feeds because of the high cost of facilities needed to achieve and maintain adequate pressure, temperature, and concentration of ammonia. In addition, it is logistically challenging, potentially hazardous for forages, and impractical to use ammoniation to detoxify large quantities of feeds for dairy farms.

#### 6.2.2. Ozonation

Ozone gas is a powerful oxidizer that can disrupt cell membranes and disperse cytoplasmic contents thus inactivating microorganisms [81]. Various studies have confirmed its efficacy in degrading aflatoxin and inactivating aflatoxin-producing fungi without affecting feed quality. de Alencar et al. [82] examined the ability of ozone to inactivate fungi and aflatoxin in peanuts and reported that 96 h of exposure to 21 mg/L of ozone gas reduced total fungal counts (cfu/g) by 3 logs, whereas 13 mg/L of ozone gas reduced fungal counts by 2 logs. Both concentrations (21 and 13 mg/L) reduced counts of *A. flavus* and *A. parasiticus* in peanuts. Likewise, Freitas-Silva and Venâncio [83] reviewed studies on the effects of ozonation in reducing aflatoxin concentration and showed that ozonation was effective in inactivating aflatoxin and other mycotoxin producing fungi (*A. fumigatus*, *A. parasiticus*, *A. niger*, *Fusarium* spp., *Alternaria* spp., *Penicillium* spp., and other *Aspergillus* spp.). Ozonation degraded aflatoxin from a variety of substrates such as cottonseed meal and flour, pistachio kernels, peanut kernels, and peanut meal [83]. Ozone treatment (21 mg/L) for 96 h reduced total aflatoxin concentration in peanuts by 30% from 190 to 140 µg/kg; however, the residual concentration was above the FDA action limit of 20 µg/kg [7]. Chen et al. [84] reported that treating peanuts contaminated with an average concentration of 200 µg/kg of total aflatoxin (containing B1, B2, G1, and G2) with 6 mg/L ozone for 30 min decreased total aflatoxins and AFB1 concentration by 65.8 and 65.9%, respectively, without affecting peanut quality. Similarly, Luo et al. [85] examined effects of 40, 60 and 90 mg/L ozone on inactivating AFB1 in naturally contaminated corn and reported that the extent of AFB1 degradation increased with increasing ozone concentration and exposure time. The authors reported 88% degradation of AFB1 with ozonation (90 mg/L; 40 min) of low moisture corn (13.5% moisture) compared with 72% for high moisture corn (20.4% moisture), without affecting corn quality. However, ozonation is not widely used to detoxify aflatoxin on dairy farms due to the same limitations as ammoniation.

#### 6.2.3. Enzyme Treatment

Degradation of aflatoxin by different types of enzymes has been previously reported in several studies [86,87] and reviewed by Loi et al. [88]. An unnamed extracellular enzyme isolated from mushrooms (*Pleurotus ostreatus*) degraded aflatoxin by cleaving the lactone ring [86]. This novel enzyme had a molecular mass of approximately 90 kDa with an optimum pH for degradation between 4.0 and 5.0 at 25 °C. Das et al. (2014) [87] showed that two strains of *P. ostreatus,* MTCC 142 and GHBBF10, degraded 0.5 ug/kg of AFB1 in rice straw by 89.14 and 91.76%, respectively. The inclusion of inorganic salts supported hyphal growth of the two strains with Cu^2+^, maximizing the degradation rate to 92.4% while other inorganic salts, such as Zn^2+^, Mg^2+^, Mn^2+^, increased the degradation rate to 82.7 to 88.5%. In addition, *P. ostreatus* MTCC 142 and *P. ostreatus* GHBBF10 showed the highest degrading ability when surfactants Triton X-100 and Tween 80 were used, respectively. The authors detected many intermediate degradation compounds of aflatoxin, suggesting sequential enzymatic conversion of bisfuran ring of AFB1 and reported that the activity of laccase and manganese peroxidase was concomitant with the aflatoxin degradation potential of the strains. Some of the other enzymes identified to possess the ability to degrade aflatoxin are laccase enzymes from white rot fungi *T. versicolor*, *A. niger*, *Streptomyces coelicor, Pleurotus pulmonarius* [89,90,91], peroxidase from *Armoracia rusticana* [92], and an enzyme with a molecular mass of 51.7 kDa, named aflatoxin-detoxifizyme, from ringless honey mushroom, *Armillaria tabescens* [93], etc.

Interestingly, fungal enzymes that degrade aflatoxin may also degrade fiber and lignin [94]. Beg et al. [95] showed that the crude protein concentration of rice husks fermented for 35 d with *P. ostreatus*, which has an efficient ligninolytic system, increased from 2.15 to 9.31% and the crude fiber concentration reduced from 40.5 to 26.2%. Consequently, the fermented rice husk had 79.4% higher reticulo-rumen digestibility than the non-fermented control. Similarly, Adamović et al. [94], reported that incubating wheat straw with *P. ostreatus* mushrooms for 120 d reduced neutral detergent fiber concentration from 82.4 to 48.5% and acid detergent fiber from 56.1 to 41.2% and these effects were attributed to *P. ostreatus* enzymes by the authors. However, feeding diets containing 0, 10 or 17% spent *Pleurotus* compost to heifers, reduced average daily gain and feed efficiency because of the lower nutritive value of and lower diet preference for the diets with the 17% inclusion level, though no adverse effects were detected with the 10% inclusion level [94]. Future studies should investigate more effective approaches to add enzymes to animal diets, such as using culture media of effective fungi or purified enzymes because of their promising effects as dual-purpose additives to degrade aflatoxin and improve fiber digestion.

#### 6.2.4. Cold Plasma

Cold plasma is a novel approach to destroy mycotoxins and microorganisms in food using cold atmospheric pressure plasma containing reactive species [96,97,98]. Cold plasma can be generated by applying a strong electric field to gases such as air, O_2_, N_2_ and He to form reactive gas species containing ions such as O^−^, OH^−^, N_2_^+^, H^+^, H_3_O^+^ and O^+^, molecular species such as N_2_, O_2_ and H_2_O_2_, as well as reactive radicals such as O•, H•, OH•, NO• [99,100]. Cold plasma degraded AFB1 into six degradation products with reduced biological activity by various potential degradation pathways including reactions involving free radicals (H•, OH•, CHO•), epoxidation by H_2_O• and oxidation of AFB1 by oxidative species.

Siciliano et al. [96] reported that 12 min of cold plasma treatment completely destroyed aflatoxin in a standard solution containing aflatoxin B1, B2, G1, and G2, and it degraded over 70% of AFB1 and total aflatoxin in hazelnuts. Several factors affected the degradation efficiency, such as gas type (pure N_2_ > 0.1% O_2_ > 1% O_2_ > 21% O_2_), power of the plasma generator (1150 > 1000 > 700 > 400 W), and exposure time (12 > 4 > 2 > 1 min). Shi et al. [97] reported degradation of AFB1 by 62 and 82% with 1 and 10 min of cold plasma treatment, respectively, at 40 % relative humidity. The degradation efficacy increased with increasing relative humidity and varied with gas type. One minute of cold plasma treatment at under 5, 40 and 80% relative humidity resulted in degradation of 66.0, 75.5 and 73.4% of AFB1, respectively, using a gas containing 65% O_2_, 30% CO_2_ and 5% N_2,_ which was less effective than a gas containing 78% N_2_ and 22% O_2_ (76 vs. 62% degradation).

The efficacy of using cold plasma to destroy mycotoxins versus other approaches, such as heat treatment, UV light irradiation, bacterial degradation, ammoniation, and ozonation, was reviewed by Hojnik et al. [99]. Compared to the other aflatoxin degrading methods, cold plasma has the advantages of high decontamination efficiency with a low energy requirement within a short process time; the process is environmentally friendly and has negligible effects on food quality [99]. However, despite its promise, no studies were found on its use for aflatoxin degradation in animal feeds, perhaps due to the cost and logistical implications. More studies are needed to establish optimum conditions for use of this promising treatment, such as processing time, gas type, and humidity. In addition, reducing the cost of plasma treatment and adapting it for large scale animal feed processing is necessary for its use in livestock production.

#### 6.2.5. Clay Sequestering Agents

Previous studies have shown that clay-based sequestering agents are effective at reducing gastrointestinal absorption of the toxin and preventing milk AFM1 concentrations from exceeding the FDA threshold [5,101,102]. These sequestering agents include several types of clay such as sodium bentonite, smectite clay, a blend of layered aluminosilicate mineral clays, and calcium montmorillonite bentonite. They can also occur in a mixture with other compounds, such as mixtures of esterified glucomannan and hydrated sodium calcium aluminosilicate (HSCAS), sodium montmorillonite with live yeast, yeast culture, mannan oligosaccharide, vitamin E, etc. A summary of commercially available products and their composition is presented in Table 3 and a list of studies that examined the effectiveness of clay and yeast based sequestering agents are shown in Table 4. Clay supplementation has shown promising results in reducing milk AFM1 and health damage caused by feeding aflatoxin-contaminated diets in many studies. However, several factors can affect their effectiveness such as the dose, relative ratio of clay to aflatoxin, particle size and mode of supplementation.

Factors affecting the efficacy of clay sequestering agents include the following:

- Inclusion Level

The efficacy of sequestering agents in reducing aflatoxin levels is dose-dependent, as shown in Table 4. Queiroz et al. [5] showed that feeding Calibrin A, a calcium montmorillonite bentonite, at 0.05% of dietary DM did not reduce AFM1 concentration in the milk of dairy cows challenged with 75 µg/kg of AFB1, but feeding it at 2% of the dietary DM reduced milk AFM1 by 16%. Similarly, Maki et al. [113] reported a linear decrease in milk AFM1 by increasing the dose of the Novasil Plus, which is a smectite bentonite form. Adding 0.58% dietary DM of Novasil Plus to dairy cow diets reduced AFM1 by 47.3%, but adding 1.17% of the sequestering agent reduced AFM1 concentration in milk by 70.9% in dairy cows challenged with 100 µg/kg of aflatoxin. However, a high dose of clay reduced milk yield in one study. Sulzberger et al. [51] showed that increasing the dose of clay (a mixture of vermiculite, nontronite, and montmorillonite) from 0.5 to 2% linearly reduced milk yield by lactating dairy cows for unknown reasons as no treatment differences in the concentrations of serum vitamin A, D and E and minerals were detected. The authors reported that mineral and vitamin concentrations in plasma were unaffected by clay consumption and they attributed the negative effects of clay on efficiency parameters to the metabolism of aflatoxin. Dietary inclusion of clay at 2% may have reduced energy utilization, as shown by the lower feed efficiency in their study. However, this is generally not a concern because dietary clay inclusion is typically about 1% or even lower [63,108]. In summary, increasing the dose of clay may reduce AFM1 contamination of milk but excessively high doses may also decrease milk yield.

- Sequestering Agent to Aflatoxin Ratio

In general, higher inclusion levels as discussed above or higher ratios of binder relative to the aflatoxin contamination level are more effective at binding aflatoxins. Xiong et al. [104] reported that dietary addition of Solis Mos (Novus International, Saint Charles, MO; a blend of sodium montmorillonite with live yeast, yeast culture, mannan oligosaccharide, and vitamin E) at 0.25% of diet DM reduced milk AFB1 by 16% in cows fed 20 µg/kg of AFB1, but did not affect milk AFM1 in cows fed 40 µg/kg of AFB1. Several studies have demonstrated that low inclusion levels of clay (<0.2 %) are not effective at reducing milk AFM1 concentration. For instance, Kissell et al. [110] and Sumantri et al. [109] observed no effects on aflatoxin levels in milk when less than 0.1% clay was included in dairy cows’ diets.

- Particle Size

Particle size may influence the effectiveness and efficiency of clay binding to aflatoxin. In the study by Katsoulos et al. [111], clinoptilolite, a natural zeolite, reduced AFM1 by 53.2% when added as larger particles (<0.8 mm) but by 58.1% when added as smaller particles (<0.15 mm). Their results suggested greater efficacy of small-particle sized clay. However, their results should be interpreted cautiously as results were compared to the day 0 pre-experimental baseline value and the concentration of AFM1 in the small particle size group was greater before the start of the experiment. Future studies are needed to definitively indicate the role of particle size in aflatoxin binding efficacy.

- Mode of Addition

Few studies have explored how the method of adding sequestering agents affects the binding efficiency to aflatoxin. Masoero et al. [108] showed that the physical process of pelletizing, which affected the sample moisture, temperature, and pressure, increased interaction between AFB1 and sequestering agents. Further, feeding a pelleted concentrate containing a commercial magnesium smectite clay reduced milk AFM1 to a greater extent compared to when the clay was added to the concentrate meal (76 vs. 111 ng/kg milk AFM1).

- *Saccharomyces cerevisiae* Based Sequestering Agents

An alternative sequestering agent, *Saccharomyces cerevisiae* fermentation product (SCFP), can adhere aflatoxin to its cell wall structure, β-glucan and mannan [116]. Yeast also has potential to improve animal performance by modulating the gut microbiome, improving gut morphology, and reducing inflammatory responses [117,118]. Several studies have confirmed the efficacy of *Saccharomyces cerevisiae* at binding AFB1 [112,119]. Shetty and Jesperson [119] reported that 7 strains of *S. cerevisiae* bound 10–20% of the AFB1 in vitro, 8 strains bound 20–40%, and 3 strains bound more than 40%. Gonçalves et al. [112] showed that the yeast cell wall and partially dehydrated yeast from the brewery industry reduced aflatoxin in milk by 69.8 and 62.8%, respectively; while autolyzed yeast and dried yeast from the sugarcane industry reduced the levels by 45.6 and 47.5% of the aflatoxin, respectively, suggesting that different yeast products have different binding efficacies.

The effectiveness of yeast products at sequestering aflatoxin are equivocal as various studies have not confirmed aflatoxin binding by yeast products [48,101,110,114]. Ogunade et al. [48] showed that feeding 0.09% dietary SCFP improved inflammation and the immune status of cows but did not reduce milk AFM1 concentration in cows challenged with 75 µg/kg AFB1. A study by Firmin et al. [120] showed that dietary inclusion of a modified yeast cell wall (0.07% in diet DM) reduced AFB1 absorption and increased AFB1 and AFM1 excretion through feces but did not reduce milk AFM1 in dairy ewes fed a diet containing 60 μg/kg AFB1.

Due to the high cost of producing yeast cell wall and fermentation products, yeast products have been generally included in diets at low (<0.3%) inclusion levels, which may partially contribute to the equivocal results of yeast in binding aflatoxin and preventing its transfer to milk. Nevertheless, yeast products are attractive for use in dairy cow diets because they are easy to apply to diets on farms of varying sizes, and they have other benefits beyond mitigating AFB1 including improving rumen function, animal performance, and health.

To improve the efficacy of yeast products, they have been combined with clay absorbents in some studies. Kutz et al. [101] showed that feeding 0.56% of dietary MTB-100, a blend of a yeast product and clay, did not affect milk AFM1 in cows challenged with 112 μg of AFB1/kg of diet DM. Weatherly et al. [114] reported that a mixture of yeast cell wall and bentonite clay supplemented at 0.13 and 0.26% of dietary DM, respectively, did not affect milk AFM1 in cows challenged with 100 µg/kg of AFB1; however, the inclusion levels of sequestering agents were low compared to 1%, which is mostly used for adding clay. Notably, Diaz et al. [103] reported a 58.5% reduction in milk AFM1 when 1.2% dietary MTB-100 was fed to cows challenged with 100 µg/kg dietary aflatoxins (55% AFB1, 40% AFG1, 2%AFB2, 3% AFG2). Therefore, the efficacy of combining yeast products with clay seems to be variable and is probably dependent on the doses and forms of the two agents.

#### 6.2.6. Lactic Acid Bacteria

Several studies have shown that lactic acid bacteria (LAB) can bind aflatoxin in vitro (Table 5). Pierides et al. [121] reported that viable or heat-killed probiotic LAB *Lactobacillus rhamnosus* strains, *Lactobacillus lactis*, *Lactobacillus gasseri*, and *Lactobacillus acidophilus* bound AFM1 in phosphate-buffered saline (PBS) solution. Subsequently, the most effective strain, *L. rhamnosus* LC-705, was reported to bind 63.6 and 69.6% of the AFM1 in skim or full cream milk, respectively, after overnight incubation. Similarly, Peltonen et al. [122] tested 12 strains of *Lactobacillus*, 5 strains of *Bifidobacterium,* and 3 strains of *Lactococcus* spp. and reported that 5.60 to 59.7% of the AFB1 was bound to the bacterial strains. *Lactobacillus amylovorus* strain CSCC5160 and CSCC 5197 and *Lactobacillus rhamnosus* strain LC1/3 were the most effective and they had bound 52.6, 66.5 and 76.9% of AFB1, respectively, after 24 h incubation in an AFB1-contaminated PBS solution. The authors attributed the binding effect to the bacterial cell wall components, such as carbohydrates and proteins, as well as the structure of the cell envelope. In a review, Shetty and Jespersen^116^ reported that the mode of binding by LAB involves physical adhesion of the toxin to the bacterial cell wall components, such as mannan.

Ma et al. [123] showed that silage inoculants based on LAB bound aflatoxin in vitro. They reported that *Lactobacillus plantarum* R2014 bound 56% of the AFB1, *Lactobacillus buchneri* R1102 bound 51.5%. and *Pediococcus acidilactici* EQ01 bound 56.9% at pH 2.5 in vitro. This binding varied with the dose and viability of the LAB as well as the prevailing pH. However, when these LAB strains were used as inoculants to ensile corn forage for 21 d, they did not sequester aflatoxin. Their findings indicated that although LAB is effective in binding aflatoxin in vitro, a more effective delivery approach is needed to ensure efficacy in the field.

No studies have examined if feeding LAB to lactating dairy cows can reduce milk AFM1. The ability of LAB to reduce aflatoxin availability in vivo is questionable because the binding is physical and reversible. Repeated washing in PBS solution can release aflatoxin that was bound to LAB [122]. In addition, LAB is normally fed in small doses, which is likely to limit colonization of the ingested aflatoxin by LAB. It would be interesting to determine if bonds between aflatoxin and LAB can be released in the gastrointestinal tract of dairy cows. Other benefits of LAB treatment of silage, such as its potential to act as a probiotic [124] and increase milk production [125] by dairy cows, highlight the importance of more work in this area, such as determining the efficacy of LAB with clay or yeast-based adsorbents.

#### 6.2.7. Chlorophyll Products and Polyphenol

- Chlorophyll Products

Studies have shown that the planar ring structure of chlorophyll allows binding of aflatoxin and reduces aflatoxin-induced damage to cells and DNA [126,127]. Hsu et al. [127] reported that chlorophyll derivatives, chlorophyllide and pheophorbide, reduced in vitro formation of an AFB1-DNA adduct in hepatoma cells, with pheophorbide being more potent than chlorophyllide. The mode of action was suggested to be direct physical trapping because pretreating cells with chlorophyll derivatives and washing them before adding AFB1 totally eliminated inhibition by chlorophyllide, and partially eliminated inhibition by pheophorbide. In addition, pheophorbide increased glutathione S-transferase activity in murine Hepa-1 cells [127], which could prevent formation of an aflatoxin-DNA adduct, facilitating the clearance of the toxin. In a double blinded trial by Egner et al. [128], consuming sodium copper chlorophyllin, a water-soluble derivative of chlorophyll reduced excretion of aflatoxin-DNA adduct repair products in individuals with a high risk for liver cancer. In addition, Simonich et al. [126] reported that feeding 250 or 300 mg/kg BW of chlorophyll and chlorophyllin, respectively, reduced hepatic DNA adduction by 42% and 55%, respectively, AFB1-albumin adducts by 65% and 71%, respectively, and the major AFB-N-7-guanine urinary adduct by over 90% in rats challenged with 250 µg/kg AFB1.

Although promising results have been shown in humans and rats, few studies have investigated the effectiveness of chlorophyll products at binding aflatoxin in ruminant animals. Ogunade et al. [48] showed that feeding chlorophyll-based sequestering agents to dairy cows challenged with 75 µg/kg DM of AFB1 did not reduce milk AFM1 concentration or improve the immune response. This may have been because the chlorophyll-based sequestering agent was dosed at a very low level, at <0.1% of the diet DM; perhaps greater inclusion levels such as 1% would increase the binding capacity of these products. More studies on the efficacy of chlorophyll products at binding aflatoxin in dairy cow diets are needed.

- Polyphenol

Several studies have demonstrated the effects of polyphenol in binding aflatoxin and reducing its damage to the health of small animals such as rats. Lu et al. [129] reported that oxidized tea polyphenols form a complex with AFB1 and inhibit the absorption of AFB1 in rats. Rats fed with 100 µg/kg BW of AFB1 and 400 mg/kg BW of oxidized tea polyphenols had lower plasma AFB1-albumin and greater fecal excretion of AFB1 excretion compared to those fed only AFB1 4 h after ingestion. Adding polyphenol also lowered liver damage as shown by the lower serum levels of alanine aminotransferase and aspartate aminotransferase compared to the levels elevated by feeding AFB1. However, the aflatoxin-mitigating potential of polyphenols has not been tested in the diet of ruminant animals; more studies are needed in this area.

#### 6.2.8. Activated Carbon

Activated carbons, also called activated charcoals, are a family of carbonaceous substances with highly porous structures developed by thermal, physical, or chemical activation processes [130]. Because of their outstanding adsorptive properties, they are used in wastewater treatment, treatment of toxic air emissions, decolorization processes, and heterogeneous catalysis [131]. Galvano et al. [131] reported that activated carbon can bind aflatoxin in vitro with higher affinity than HSCAS. However, only a few in vivo studies have examined the effectiveness of activated carbon at reducing AFB1 transfer to milk, and the results are equivocal. Galvano et al. [132] reported that in lactating cows fed 11.28 μg AFB1 /kg of diet, 2% of a dietary activated carbon sorbent was more effective than 2% of HSCAS or 2% of another activated carbon at reducing milk AFM1, resulting in reduction by 45.3, 32.5 and 22.0%, respectively. Rao and Chopra [133] showed that 1% dietary activated charcoal and 1% dietary sodium bentonite reduced milk AFM1 by 66.6 and 76.0% compared to milk AFM1 levels in goats fed 100 µg/kg DM of AFB1. However, Diaz et al. [103] reported that feeding activated carbon at 0.25% of dietary DM did not affect milk AFM1 in dairy cows challenged with 55 µg/kg total aflatoxins. This may have been due to the low dose used. More studies are needed to document the efficacy of mitigating dietary aflatoxin in dairy cow diets with activated charcoal or carbon.

#### 6.2.9. Antioxidants

Aflatoxin ingestion can cause oxidative damage to animals by inducing overproduction of ROS and reducing concentrations of non-enzymatic antioxidant, such as glutathione and ascorbic acid [104,134,135]. Many studies have reported that antioxidants reduced aflatoxin damage in vitro, in mice and ruminant animals [134,135]. Alpsoy et al. [135] revealed that 1560 µg/L of aflatoxin reduced the glutathione (an antioxidant) level and superoxide dismutase activity of human lymphocytes, and adding vitamin A, C, or E restored the levels to the normal range by inhibiting ROS generation. Verma and Nair [134] reported that 25 or 50 g/d of aflatoxins (B1, B2, G1, G2 in the ratio of 8:3:2:1) induced dose-dependent increases in lipid peroxidation in the testes of mice. They also reported that aflatoxin reduced levels of non-enzymatic (glutathione and ascorbic acid) and enzymatic (superoxide dismutase and glutathione peroxidase) antioxidants, and these effects were partially inhibited by feeding 2 mg/d of vitamin E.

The combination of clay sequestering agents and antioxidants may improve aflatoxin binding and animal health. Xiong et al. [104] reported that feeding a mixture of sodium montmorillonite with live yeast, yeast culture, mannan oligosaccharide, and vitamin E to cows challenged with 20 µg/kg DM AFB1 reduced the transfer of aflatoxin to milk and improved antioxidative status by increasing the plasma superoxide dismutase concentration and reducing malondialdehyde, which is a lipid peroxidation product that can indicate oxidative stress. In addition, the dietary inclusion of 0.36% Unike Plus (Nutriad Animal Feed Additives, Dendermonde, Belgium), a mixture of adsorbent clay minerals, inactivated yeast (*S. cerevisiae*), undisclosed botanical components, antioxidants, and preservatives, reduced AFM1 by 52% in cows consuming 105 µg/kg DM of the mixed aflatoxins [52]. However, the effects of antioxidants are not clear due to the lack of measurement of oxidative stress related parameters. Because of the well-known effects of aflatoxin on inducing oxidative stress, the inclusion of antioxidants in some sequestering agents may have protective effects against oxidative stress. However, their effects on dairy cows undergoing aflatoxin challenge are not clear due to lack of data; more research is needed in this area.

Future studies should focus on developing cost-effective approaches that can be implemented on farms to detoxify aflatoxins in feeds before they are fed to animals. In addition, more research on next-generation sequestering agents that bind a variety of mycotoxins and improve animal performance is warranted.

## 7. Conclusions

Aflatoxin can negatively impact the production, immunity, health, and reproduction of ruminant animals. Therefore, preventing aflatoxin contamination pre-harvest and destroying the toxin after harvesting and during storage are critical to safeguard animal and human health and welfare to maintain the profitability of dairy production. However, the existing AFB1 detoxification methods are not applicable on dairy farms. Therefore, when prevention of aflatoxin contamination of feeds in the field or during storage fails, the most effective option is to add AFB1 sequestering agents to the diets. Though several options exist, most have not had consistent effects, with the exception of clay-based products. More research is needed on effective, economical methods of detoxifying or sequestering AFB1 in dairy cow diets.

## Figures and Tables

**Table 1:** Summary of aflatoxin contamination for different regions in the world from samples collected during 2018 (based on the Biomin mycotoxin survey, 2018 [35]).

		Europe	Middle East	Africa	Asia	North America	South and Central America
Finished Feed	No. of samples	1146	87	161	1458	537	1033
	% of samples positive for aflatoxin	12%	11%	11%	44%	12%	25%
	Average of positive samples, µg/kg	5	3	9	26	9	9
	Median of positive, µg/kg	2	2	4	8	4	4
	Maximum, µg/kg	136	15	26	697	57	216
Corn	No. of samples	371	14	191	685	478	3656
	% of samples positive for aflatoxin	18%	29%	3%	37%	9%	19%
	Average of positive samples, µg/kg	9	2	3	42	36	11
	Median of positive, µg/kg	2	1	2	8	15	4
	Maximum, µg/kg	76	6	8	636	280	402
Cereals ^1^	No. of samples	743	8	28	267	48	586
	% of samples positive for aflatoxin	11%	13%	21%	13%	6%	53%
	Average of positive samples, µg/kg	2	2	8	13	6	5
	Median of positive, µg/kg	1	2	4	1	7	4
	Maximum, µg/kg	19	2	27	88	8	40

^1^ wheat, barley, oats, rice, sorghum, millet.

**Table 2:** Summary of aflatoxin contamination in different regions of the world from samples collected during 2019 (based on the Biomin mycotoxin survey, 2019 [36]).

		Europe	Middle East and North Africa	Africa	Asia	North America	South and Central America
Finished Feed	No. of samples	1042	84	326	1589	690	1530
	% of samples positive for aflatoxin	8%	2%	25%	30%	6%	28%
	Average of positive samples, µg/kg	10	42	33	19	11	5
	Median of positive, µg/kg	4	12	13	8	5	4
	Maximum, µg/kg	237	615	370	430	94	134
Corn	No. of samples	427	30	376	717	524	4091
	% of samples positive for aflatoxin	9%	37%	7%	31%	4%	21%
	Average of positive samples, µg/kg	8	2	16	43	132	10
	Median of positive, µg/kg	4	1	6	10	5	4
	Maximum, µg/kg	54	5	64	773	1327	1264
Cereals ^1^	No. of samples	766	3	20	90	64	375
	% of samples positive for aflatoxin	21%	0%	5%	12%	3%	54%
	Average of positive samples, µg/kg	2	-	1	13	5	4
	Median of positive, µg/kg	2	-	1	4	5	2
	Maximum, µg/kg	6	0	1	68	7	30

^1^ wheat, barley, oats, rice, sorghum, millet.

**Table 3:** Commercially available aflatoxin-sequestering agents.

Name	Company	Compounds	Reference
Astra-Ben 20	Prince AgriProducts, Quincy, IL	Sodium bentonite	Diaz et al., 2004 [103]
Flow Guard	Laporte Biochem, Inc., Milwaukee, WI	Sodium bentonite	Diaz et al., 2004 [103]
Mycosorb	American Colloid Co., Arlington Heights, IL	Sodium bentonite	Diaz et al., 2004 [103]
Red Crown bentonite	Prince AgriProducts, Quincy, IL	Bentonite	Diaz et al., 2004 [103]
SA-20	Westvaco, Covington, VA	Activated Carbon	Diaz et al., 2004 [103]
Calibrin A	Amlan International, Chicago, IL	Calcium montmorillonite bentonite	Queiroz et al., 2012 [5]
MTB-100	Alltech Inc., Nicholasville, KY	Esterified glucomannan with HSCAS	Kutz et al., 2009 [101]
NovasilPlus	BSAF, Ludwigshafen, Germany	Smectite clay	Kutz et al., 2009 [101]
Solis	Novus International, Saint Charles, MO	A blend of layered aluminosilicate mineral clays	Kutz et al., 2009 [101]
Solis Mos	Novus International, Saint Charles, MO	Sodium montmorillonite with live yeast, yeast culture, mannan oligosaccharide, and vitamin E	Xiong et al., 2015 [104]
Toxy-Ni	Nutriad Animal Feed Additives, Dendermonde, Belgium	Adsorbent clay minerals and inactivated yeast (*Saccharomyces cerevisiae*)	Rodrigues et al., 2019 [52]
Unike Plus	Nutriad Animal Feed Additives, Dendermonde, Belgium	Adsorbent clay minerals, inactivated yeast (*S. cerevisiae*), botanical components, and a mixture of antioxidants and preservatives	Rodrigues et al., 2019 [52]
Mycofix Plus	Biomin GmbH, Herzogenburg, Australia	Bentonites, enzymes, *Eubacterium* strain (BBSH 797), and yeast strain *T. mycotoxinivorans*	Pietri et al., 2009 [105]
FloMatrix	PMI nutritional Additives, Arden Hills, MN	Aluminosilicate clay matrices and yeast components	Pate et al., 2018 [106]

**Table 4:** Efficacy of sequestering agents at reducing milk AFM1 in dairy cows—a summary of controlled studies published from 1991 to 2020.

Study	Aflatoxin Dose in Diet	Sequestering Agents	% of Sequestering Agents as Diet DM	% Reduction of Milk AFM1
Harvey et al., 1991 [107]	200 µg/kg AF	HSCAS	0.5%	24%
	100 µg/kg AF	HSCAS	1%	44%
Diaz et al., 2004 [103], Exp 1	100 µg/kg total AF, 55% AFB1; 40% AFG1; 2% AFB2 and 3% AFG2	Astra-Ben 20 ^1^	1.2%	61%
		FlowGuard	1.2%	65%
		Mycrosorb	1.2%	50%
Diaz et al., 2004 [103], Exp 2	100 µg/kg total AF. 55% AFB1; 40% AFG1; 2% AFB2 and 3% AFG2	Astra-Ben 20 ^1^	1.2%	64.4%
		Red Crown bentonite	1.2%	31.4%
		MTB-100 ^1^	0.05%	58.5%
		Activated Carbons	0.25%	5.4%
Masoero et al., 2008 [108]	7.4 µg/kg AFB1 Exp 1	Magnesium smectite clay (Atox)	0.82%	47.4%
	7.5 µg/kg AFB1 Exp 2	AFB1-contaminated complete concentrate with magnesium smectite clay as a pellet	0.83%	76 ng/kg AFM1 in milk
		AFB1-contaminated complete concentrate with magnesium smectite clay as a meal	0.83%	111 ng/kg AFM1 in milk
Kutz et al., 2009 [101]	100 µg/kg AFB1, a mixture of AFs contains 61% AFB1, 2% AFB2, 26% AFG1, 1% AFG2	Solis ^1^	0.56%	44.8%
		NovasilPlus ^1^	0.56%	47.9%
		MTB-100 ^1^	0.56%	4.2% (NS)
Pietri et al., 2009 [105]	97.3 µg/kg AFB1	Mycofix Plus ^1^	0.08%	31%
		Mycofix Plus ^1^	0.2%	41%
Queiroz et al., 2012 [5]	75 µg/kg AF, 64% AFB1, 2% AFB2, 33% AFG1, and 0.003% AFG2	Calibrin A ^1^	0.05%	−22% (NS)
		Calibrin A ^1^	2%	16%
Sumantri et al., 2012 [109]	30.8 µg/kg AFB1	Bentonite (type not described)	0.005%	1.7% (NS)
		Bentonite (type not described)	0.045%	9.6% (NS)
Kissell et al., 2013 [110] Exp1	91 µg/kg AFB1	Experimental product (yeast cell wall extract. glucomannan) and aluminosilicate (Lallemand)	0.04%	−5.2% (NS)
Exp 2	94 µg/kg AFB1	MTB-100-2004, formulation of 2004	004%	−8.0% (NS)
		MTB-100-2006, formulation of 2006	0.04%	−6.2% (NS)
		Experimental product (Alltech)	0.04%	−9.5% (NS)
Exp 3	86 µg/kg AFB1	MTB-100-2006 ^1^	0.2%	−9.5% (NS)
		Astra-Ben 20 ^1^	0.9%	60.4%
Xiong et al., 2015 [104] Exp1	20 µg/kg of AFB1	Solis Mos ^1^	0.25%	16%
Exp2	40 µg/kg of AFB1	Solis Mos ^1^	0.25%	2% (NS)
Maki et al., 2016 [102]	100 µg/kg AF, 79% AFB1, 16% AFG1, 4% AFB2, and 1% AFG2.	NovasilPlus ^1^	0.58%	47.3%
		NovasilPlus ^1^	1.17%	70.9%
Katsoulos et al., 2016 [111]	Not a feeding trial, data is from 15 commercial herds with milk AFM1> 0.05 µg/kg	Clinoptilolite, a natural zeolite, particle size <0.15mm	1%	58.1%
		Clinoptilolite, a natural zeolite, particle size <0.8 mm	1%	53.2%
Ogunade et al., 2016 [48]	75 µg/kg AFB1	SCFP with low dose chlorophyll-based additive (Diamond V)	0.09%	−0.01% (NS)
		SCFP with high dose of chlorophyll-based additive (Diamond V)	0.09%	0% (NS)
		Low dose of the additive and sodium bentonite clay (Diamond V)	0.05%	−0.01% (NS)
Gonçalves et al., 2017 [112]	480 µg/d AFB1, intake not reported	Yeast cell wall (ICC Brazil)	20 g/cow/d	69.4%
		Autolyzed yeast from sugarcane industry (ICC Brazil)	20 g/cow/d	45.6%
		Dried yeast from sugarcane industry (ICC Brazil)	20 g/cow/d	47.5%
		Partially dehydrated yeast from brewery industry (ICC Brazil)	20 g/cow/d	62.8%
Sulzberger et al., 2017 [51]	100 µg/kg AFB1, 71% AFB1, 2.5% AFB2, 24.8% AFG1, 0.006% AFG2	Clay (composition not described, UMG Minerals Group)	0.5%	18.6%
		Clay, (UMG Minerals Group)	1%	30.2%
		Clay, (UMG Minerals Group)	2%	41.9%
Maki et al., 2017 [113]	50 µg/kg AFB1	Novasil Plus ^1^	0.125%	17.3%
		Novasil Plus ^1^	0.25%	22.7%
		Novasil Plus ^1^	0.5%	71%
Pate et al., 2018 [106]	100 µg/kg AFB1	FloMatrix ^1^	0.4%	NS
		FloMatrix ^1^	0.8%	NS
Weatherly et al., 2018 [114]	100 µg/kg AFB1	Yeast cell wall and bentonite clay (Biorigin)	0.13%	−3.9% (NS)
		Yeast cell wall and bentonite clay (Biorigin)	0.26%	−4.6% (NS)
		A prototype adsorbent (Biorigin)	0.26%	−8.8% (NS)
Xiong et al., 2018 [104]	20 µg/kg AFB1	Solis Mos ^1^	0.25%	31.6%
Rodrigues et al., 2019 [52]	105.5 µg/kg AF, 72.2% AFB1, 2.4% AFB2, 24.8% AFG1, 0.6% AFG1	Toxy-Ni ^1^	0.4%	66.7%
		Unike Plus ^1^	0.4%	50%
Gallo et al., 2020 [54]	17.53 µg/kg AFB1	Smectite clay	0.5%	64.8%
Intanoo et al., 2020 [115]	22.28 µg/kg AFB1	*K. marxianus* CPY1	Total 2 g, 1 × 10^9^ CFU/g	76.6%
		*K. marxianus* RSY5	Total 2 g, 1 × 10^9^ CFU/g	72.1%
		*P. kudriavzevii* YSY2	Total 2 g, 1 × 10^9^ CFU/g	66.9%

^1^ Composition of sequestering agents listed in Table 3. NS: not significant.

**Table 5:** Some microbial strains that bound aflatoxin in previous studies.

Approach	Most Effective Strains Identified	Maximum Binding Efficiency	Factors Affecting Binding Efficiency	Reference
In vitro	*S. cerevisiae* strain A18	69.1%	Strain, temperature, acidity environment, incubation time, aflatoxin concentration, growing phase of bacteria	Shetty and Jesperson (2007) [119]
In vitro	Dead *L. buchneri* R1102 Dead *L. plantarum* R2014 *P. acidilactici* EQ01	66.5% 60.5%, 56.9%	Dose, viability, bacteria species and pH	Ma et al. (2017) [123]
In vitro	*L. rhamnosus* strain GG *L. rhamnosus* strain LC-705	77% 75%	Strain Viability	Pierides et al. (2000) [121]
In vitro	*L. amylovorus* strain CSCC5160	73.2%	Stain and incubation time	Peltonen et al. (2001) [122]
	*L. amylovorus* CSCC 5197	72.4		
	*Lactobacillus rhamnosus* strain LC1/3	76.9		

## Data Availability

Data sharing not applicable.
